# Correlative light and electron microscopic observation of calcium phosphate particles in a mouse kidney formed under a high-phosphate diet

**DOI:** 10.1038/s41598-023-28103-3

**Published:** 2023-01-16

**Authors:** Batpurev Battulga, Kazuhiro Shiizaki, Yutaka Miura, Yasuyuki Osanai, Reiji Yamazaki, Yoshiaki Shinohara, Yoshiyuki Kubota, Toru Hara, Makoto Kuro-o, Nobuhiko Ohno

**Affiliations:** 1 Division of Histology and Cell Biology, Department of Anatomy, Jichi Medical University, Shimotsuke, Japan; 2 Division of Anti-Aging Medicine, Center for Molecular Medicine, Jichi Medical University, Shimotsuke, Japan; 3 Section of Electron Microscopy, Supportive Center for Brain Research, National Institute for Physiological Sciences, Okazaki, Japan; 4 Department of Physiological Sciences, The Graduate University for Advanced Studies (SOKENDAI), Okazaki, Japan; 5 Support Unit for Electron Microscopy Techniques, Research Resources Division, RIKEN Center for Brain Science, Wako, Japan; 6 Electron Microscopy Analysis Station, Research Network and Facility Service Division, National Institute for Materials Science, Tsukuba, Japan; 7 Division of Ultrastructural Research, National Institute for Physiological Sciences, Okazaki, Japan

**Keywords:** Scanning electron microscopy, Calcification, Phosphorus metabolism disorders

## Abstract

Calcium phosphate forms particles under excessive urinary excretion of phosphate in the kidney. While the formation of calcium phosphate particles (CaPs) has been implicated in the damage to renal tubular cells and renal dysfunction, clarifying the ultrastructural information and the elemental composition of the small CaPs in the wide areas of kidney tissue has been technically difficult. This study introduces correlative and sequential light as well as electron microscopic CaP observation in the kidney tissue by combining fluorescent staining for CaPs and energy-dispersive X-ray spectroscopy (EDS) in scanning electron microscopy (SEM) on resin sections prepared using high-pressure freezing and freeze substitution. CaPs formed in mouse kidneys under long-term feeding of a high-phosphate diet were clearly visualized on resin sections by fluorescence-conjugated alendronate derivatives and toluidine blue metachromasia. These CaPs were verified by correlative observation with EDS. Furthermore, small CaPs formed in the kidney under short-term feeding were detected using fluorescent probes. The elemental composition of the particles, including calcium and magnesium, was identified following EDS analyses. These results suggest that the correlative microscopy approach is helpful for observing in situ distribution and elemental composition of CaPs in the kidney and contributing to studies regarding CaP formation-associated pathophysiology.

## Introduction

Calcium phosphate is abundant in bony tissues; however, the deposition and accumulation of calcium phosphate salts have been observed in different organs under pathological conditions^1,2^. Calcium phosphate deposition can be formed as calcium phosphate particles (CaPs) in the kidney; the formation was caused by excessive urinary excretion of phosphate and worsened the damage to renal tubular cells^3,4^. Moreover, CaPs are involved in subepithelial or interstitial plaques in the renal papilla, which are called Randall’s plaques, and are considered to contribute to nephrolithiasis development^5^. Small CaPs can be formed from amorphous calcium phosphate and develop into larger CaPs through calcium phosphate crystallization^6,7^. CaP development is inhibited by several mechanisms, including fetuin-A in the plasma that binds to calcium phosphate precipitates and prevents them from growing into huge crystals in the extracellular space, and magnesium, which would prevent physicochemical and active cellular mechanisms^6,8–11^. The observation of newly formed small CaPs with their composition and structural environment would provide information to elucidate the mechanisms and roles associated with the formation, development, and fate of CaPs in kidney tissues. However, owing to the technical limitations of the available methods, the microscopic observation of tissue architecture and cellular ultrastructure as well as detailed elemental analyses of small CaPs in large kidney areas have been difficult.

Multiple imaging methods aided the achievement of the observation of CaPs in cells and tissues. In vivo detection methods for calcium phosphate deposition at the whole-body level include micro-computed tomography; fluorescence molecular tomography with far-red OsteoSense, which is a synthetic bisphosphonate derivative that is conjugated with a far-red fluorescent dye; and single-photon emission computed tomography with technetium-99 m methylene diphosphonate^12–14^. Furthermore, the ex vivo imaging of CaPs was performed using light microscopic observation of OsteoSense staining, and bisphosphonate derivatives for light microscopic imaging were conjugated with various fluorescent dyes for multicolor imaging^15,16^. Alternatively, elemental analyses, including those using energy-dispersive X-ray spectroscopy (EDS) in scanning electron microscopy (SEM), are well-established methods for detecting CaPs. These methods have been helpful in clarifying ultrastructure in addition to the elemental CaP composition in cultured cells and tissues^17,18^. However, the regions that can be analyzed in electron microscopy are generally smaller than those in the other methods. Additionally, the use of heavy metals for electron microscopic staining, including lead, could impair CaP detection because of the ion exchange mechanisms^19^. Therefore, whether a combination of the available methods would establish a workflow that allows efficient microscopic CaP detection and detailed ultrastructural and elemental analyses of those CaPs in large areas of tissue specimens remain unclear.

In this study, correlative and sequential light and electron microscopic observation of CaPs, which is called “CaP-CLEM,” in kidney tissues was introduced by combining (1) high-pressure freezing (HPF) followed by freeze substitution (FS), (2) light microscopic observation of CaPs labeled with alendronate conjugated with a fluorescent dye, and (3) electron microscopic observation of elemental composition with tissue ultrastructure together with EDS in SEM with highly sensitive backscattered electron (BSE) detectors. This method was applied to observe CaPs formed under long- or short-term feeding with a high-phosphate diet (HPD) and visualized the elemental composition of large and small CaPs in kidney tissues in situ. Our results suggest that CaP-CLEM is a useful approach to visualize small and large CaPs in tissue sections and allow detailed analyses of their distribution and elemental compositions in the complex histological architecture of kidney tissues.

## Results

**Figure 1:**
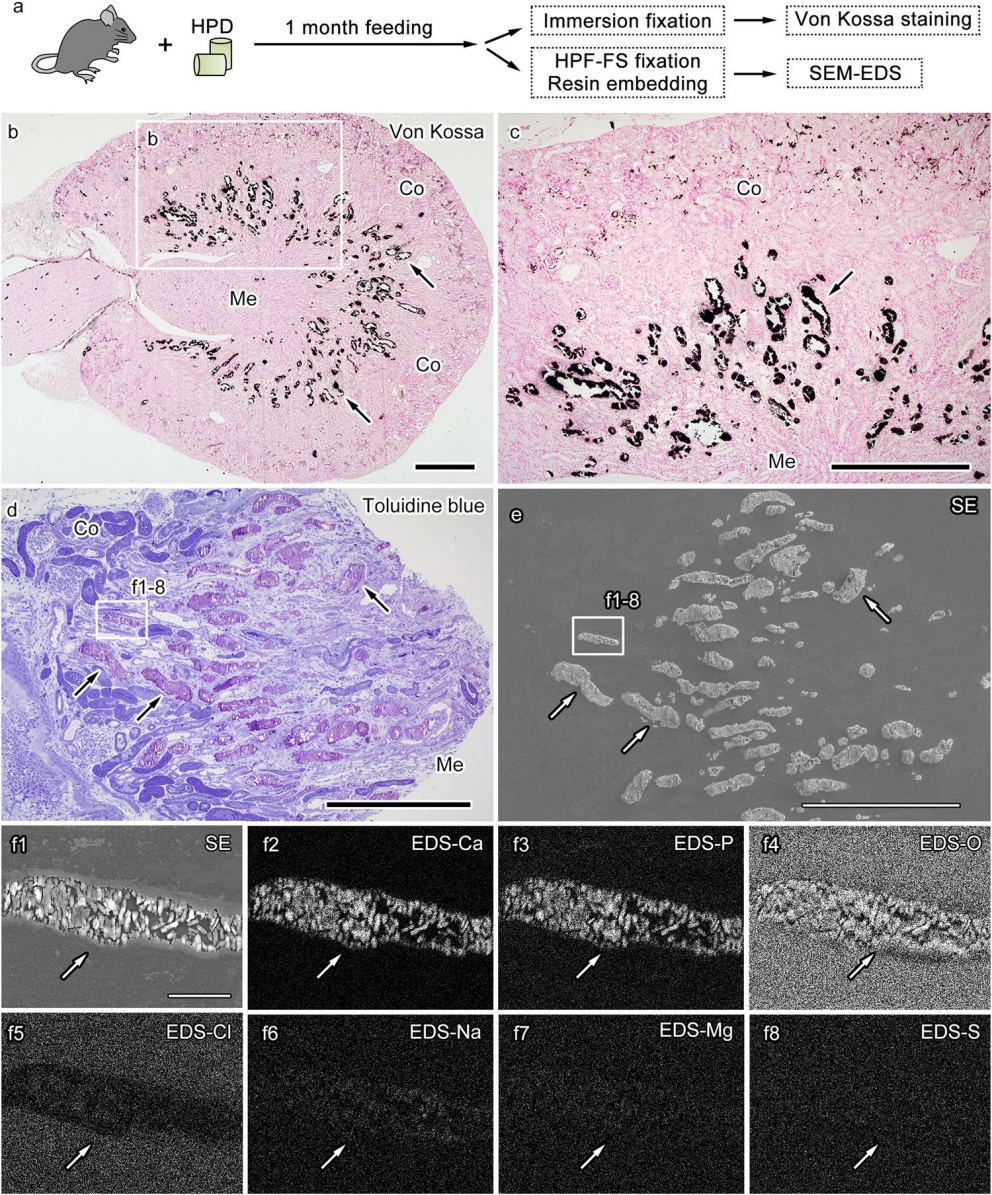
Calcium phosphate particle (CaP) detection in the corticomedullary junction of the kidney under a 4-week high-phosphate diet (HPD). Schematic drawing of the experimental design, including high-pressure freezing followed by freeze substitution (HPF-FS) and scanning electron microscopy (SEM) with energy-dispersive X-ray spectroscopy (EDS) (**a**). A representative image of an HPD-fed mouse kidney section stained using von Kossa staining (**b**). The marked area (**b**) is magnified (**c**) in von Kossa staining, and silver salt is detected in the corticomedullary junction between the renal cortex (Co) and medulla (Me) (**b**,**c**, arrows). A semi-thin resin section of the corticomedullary junction stained with toluidine blue (**d**) shows profiles with metachromasia (**d**, arrows) in areas between Co and Me. The corresponding profiles are indicated in the secondary electron image (SE) obtained from the serial section (**e**, arrows). In the area marked with a rectangle (**d**,**e**), the EDS analyses of the profile (**f1**, arrow) at a higher magnification demonstrating calcium (Ca,** f2**, arrow), phosphorus (P,** f3**, arrow), and oxygen (O,** f4**, arrow) signals, although little signals of chlorine (Cl,** f5**, arrow), sodium (Na, f6, arrow), magnesium (Mg,** f7**, arrow), and sulfur (S,** f8**, arrow). Scale bars: 500-μm (**b**–**e**) and 30-μm (**f1**–**f8**).

To develop a method for the efficient detection of CaPs in kidney tissues, first, a mouse model, where CaP formation can be observed in the kidney, was used by feeding mice with HPD for a prolonged period^20^. In this model, the mice were fed using HPD for 1 month, and their kidney tissues were dissected out, cut into small pieces, and fixed with either immersion fixation or HPF followed by FS (Fig. 1a). HPF followed by FS is considered beneficial for maintaining cell and tissue morphology and CaPs in cells and tissues^17,21^. In kidney tissues with prolonged HPD feeding, calcium phosphate deposition, which was visualized using von Kossa staining, was clearly detected and largely distributed at the corticomedullary border of the kidney (Fig. 1b,c). In the thick sections of the tissues fixed with HPF and embedded in epoxy resin, toluidine blue staining on semi-thin sections clearly revealed that several areas in the corticomedullary border contained profiles showing metachromasia, corresponding to the characteristic changes in staining colors induced by the interactions between the staining dye and stained components (Fig. 1d)^22^. When the same areas of their serial sections were observed using SEM and EDS, the profiles with metachromasia corresponded to CaPs, which could be verified in the elemental analyses with EDS and clearly included elements of calcium, phosphorus, and oxygen although not chlorine, sodium, magnesium, or sulfur (Fig. 1e,f1–f8). In contrast, no CaPs were observed in the control mice fed with a normal diet (data not shown). These results indicate that large calcium phosphate depositions, which were formed in the corticomedullary border of kidney tissues in mice after long-term feeding with HPD, are visible with metachromasia in toluidine blue staining and dense labeling in von Kossa staining and verified using EDS in the observation of serial sections.

**Figure 2:**
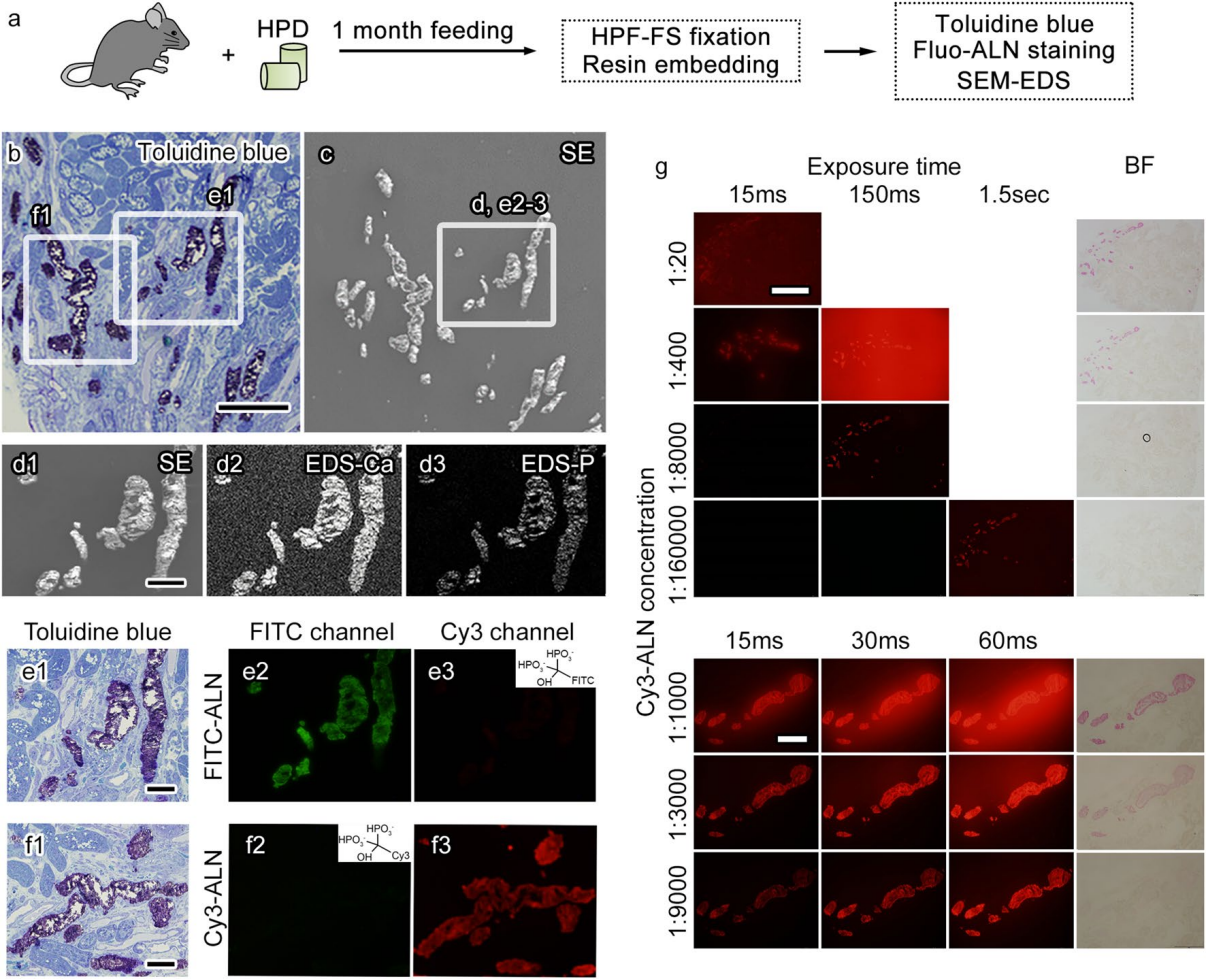
Histological detection of calcium phosphate particles (CaPs) by staining with fluorescence-conjugated alendronate derivatives (Fluo-ALN). A scheme of experiments (**a**) indicating that the semi-thin sections of kidney tissues prepared by high-pressure freezing followed by freeze substitution (HPF-FS) and embedded in resin are stained with toluidine blue or Fluo-ALN and observed with light microscopy or scanning electron microscopy (SEM) with energy-dispersive X-ray spectroscopy (EDS). Images of serial sections stained with toluidine blue and observed using light microscopy (**b**) or observed with SEM–EDS (**c**, **d**) for a secondary electron image (**d1**, SE) or signals of calcium (**d2**, EDS-Ca) and phosphorus (**d3**, EDS-P). The marked areas (**b**) are magnified (**e1**,**f1**). The marked areas (**c**) are magnified (**d**,**e2**,**e3**) in the same section with FITC-conjugated Fluo-ALN (FITC-ALN, inset of **e3**). Light microscopy images indicate that the CaPs with metachromasia (**e1**) are observed in the green channel (**e2**) although not in the red channel (**e3**) in the staining with FITC-ALN (inset of **e3**). The CaPs with metachromasia (**f1**) are observed in the red channel (**f3**) although not in the green channel (**f2**) in the serial sections stained using Cy3-conjugated Fluo-ALN (Cy3-ALN, inset of **f2**). The resin sections stained with Cy3-ALN at various concentrations are observed with light microscopy with the variable exposure time (**g**). Values on the left side indicate the Cy3-ALN concentrations. Scale bars: 200-μm (**b**), 50-μm (**d**–**f**), 500-μm (**g**, upper images), and 100-μm (**g**, lower images).

Next, developing a method for detecting CaPs that can be observed in EDS on resin sections and applicable to a correlative observation was sought. For this purpose, a study using probes of alendronate derivatives conjugated with fluorescent dyes^15^, which bind to calcium phosphate and are detectable in light microscopy observation, was conducted^14^. To facilitate subsequent analyses of tissue structures and elemental composition, the application of these probes in the analyses of sections prepared with HPF and embedded in resin was sought. In the kidney tissue obtained from mice under prolonged HPD feeding, CaPs, which were visible with toluidine blue staining in serial sections, were clearly labeled with the probes, which were conjugated with either green (FITC) or red (Cy3) fluorescent dyes and verified using EDS in the same or serial sections of the kidney tissue (Fig. 2a–c,d1–d3,e1–e3). The probes’ fluorescence distribution labeling CaPs was considered specific to the CaPs since red and green background fluorescence was negligible in staining with green and red probes, respectively (Fig. 2e1–e3,f1–f3). Alternatively, staining with multiple concentrations of the fluorescent alendronate derivative probe indicates that the fluorescence intensity seems dependent on the concentration and imaging conditions (Fig. 2g). The staining can be dimmed and become visible even in bright-field observation when the concentration is high, suggesting that suboptimal staining conditions impair CaP detection on resin sections, and the optimal staining and observation conditions of Cy3-ALN appeared to be 1/3000 concentration and 30 ms exposure time. These results show that correlative or sequential light and electron microscopic observation with the fluorescent probe of alendronate derivatives and EDS in SEM detects CaPs and allows elemental analyses in kidney tissues containing large CaPs induced by prolonged HPD feeding.

**Figure 3:**
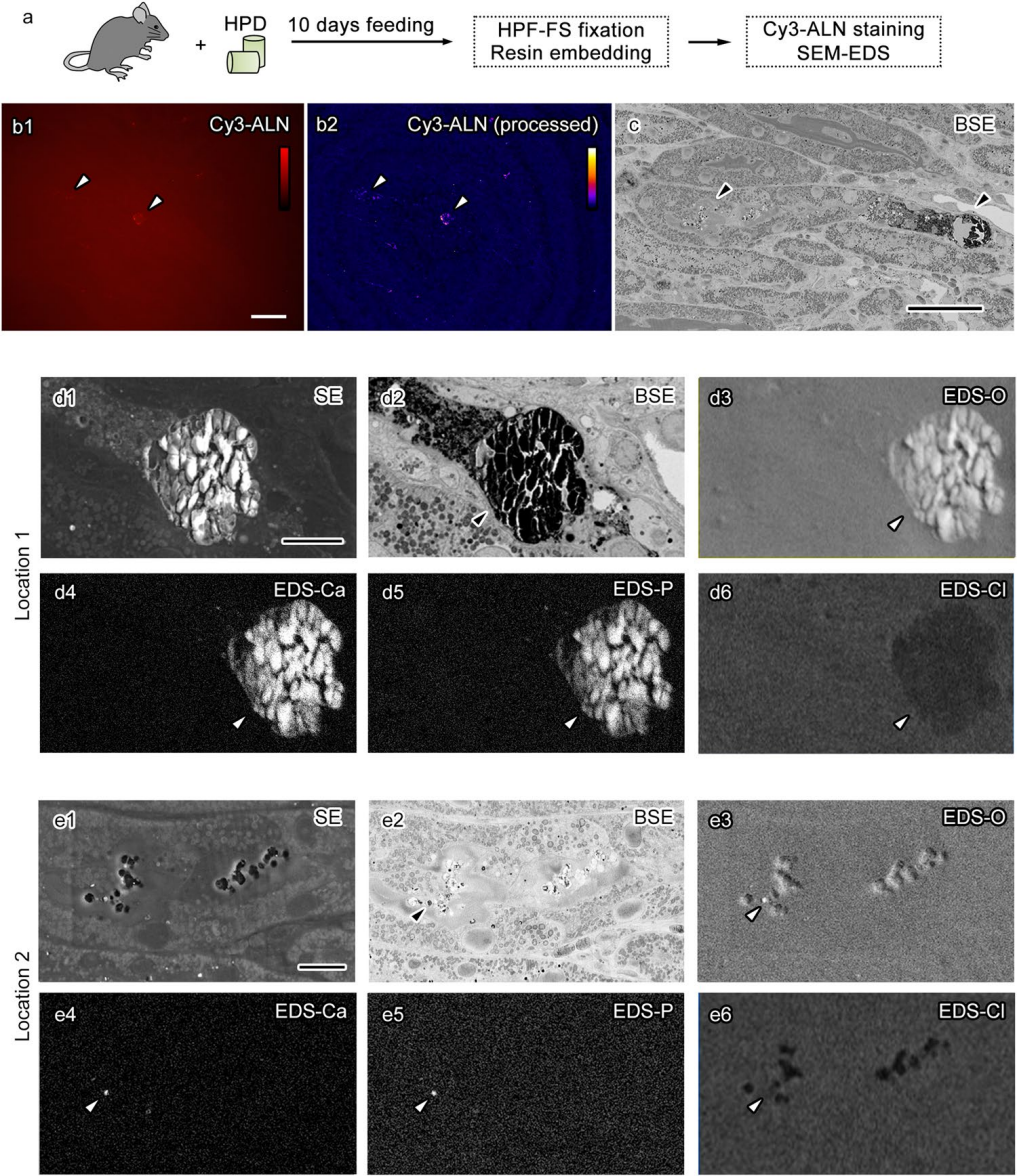
Histological detection of small calcium phosphate particles (CaPs) formed under 10 days of high phosphate diet (HPD) using a combination of light and electron microscopy. Schematic drawing of the experimental design (**a**). Light microscopy images of small positive signals (arrowheads) on a semi-thin resin section stained using Cy3-conjugated alendronate derivatives (Cy3-ALN) are indicated with (**b2**) or without (**b1**) image processing, including background subtraction, linear enhancement, and lookup table modification. The same locations (**c**, arrowheads) are observed using scanning electron microscopy (SEM) with energy-dispersive X-ray spectroscopy (EDS) for a backscattered electron image (BSE, **c**). The indicated locations (**c**, arrowheads) are observed to show secondary electron images obtained using the Everhart–Thornley detector (ETD, **d1**,**e1**) in addition to BSE (**d2**,**e2**) and EDS (**d3**–**d6**,**e3**–**e6**) images at higher magnification. The magnified images show two particles (**d**,**e**, arrowheads) with signals of oxygen (EDS-O, **d3**,**e3**), calcium (EDS-Ca, **d4**,**e4**), and phosphorus (EDS-P, d5, **e5**) although not chlorine (EDS-Cl, **d6**,**e6**). Scale bars: 50-μm (**b**,**c**) and 10-μm (**d**,**e**).

**Figure 4:**
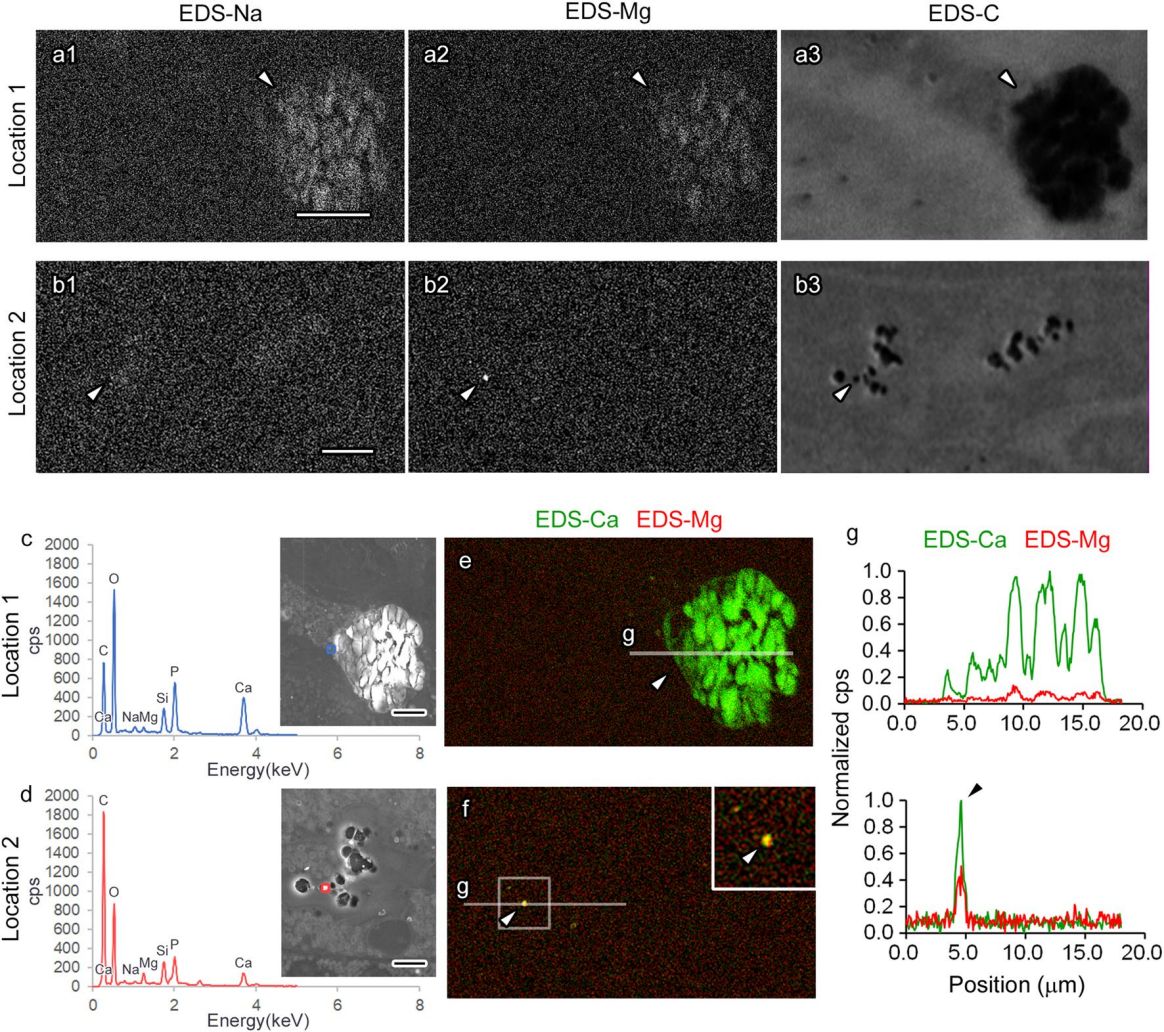
Differential composition of elements among calcium phosphate particles (CaPs) formed in the kidney under a short-term high phosphate diet (HPD). The energy-dispersive X-ray spectroscopy (EDS) images of the two CaPs (**a**,**b**) indicated in Fig. 3d,e, respectively, demonstrate the intensities of sodium (EDS-Na, **a1**,**b1**), magnesium (EDS-Mg, **a2**,**b2**), and carbon (EDS-C, **a3**,**b3**). In the EDS spectrum of the particles (**c**,**d**, blue or red line), the peaks of some elements, including magnesium, appear different (**c**,**d**). In the images where EDS-Ca and EDS-Mg signals are colored green and red, respectively, and merged (**e**,**f**), the line plots (**g**, correspond to the white lines in **e** and **f**) on the particles (**e**,**f**, arrowheads) indicate that the magnesium intensity in the small particle is higher than the adjacent background pixels (**f**,**g**, arrowheads). Scale bars: 10-μm (**a**,**b**) or 5-μm (**c**,**d**).

To assess the observation methods combining light and electron microscopy on resin sections for detecting smaller and fewer CaPs, resin-embedded kidney tissues which were obtained from mice fed with HPD for a shorter time was examined (Fig. 3a). Previous studies suggested that CaPs could be formed and detected in urine and urinary tubules; however, a clear CaP formation was undetectable on tissue sections using von Kossa staining^4,20^. In those kidney tissues, the initial trial to visualize CaPs using metachromasia in toluidine blue staining was unsuccessful (data not shown), presumably owing to the insufficient difference in staining color or the lack of metachromasia in small CaPs. By contrast, staining using the fluorescent alendronate derivative probe conjugated with Cy3 visualized small CaPs in the kidney tissue, whose fluorescent signals became clearer with the image processing, including background subtraction and lookup table adjustment (Fig. 3b1–b2). The observation of the same region of interest in serial sections using SEM and EDS clearly visualized small CaPs, whose sizes appeared as small as 1 μm. Additionally, they were observed in the tubular lumens of the kidney tissue (Fig. 3c,d1–d6,e1–e6). Elemental analyses using EDS showed the enrichment of calcium, phosphorus, and oxygen although not chlorine, and confirmed the localization of CaPs in these regions. In the detailed analyses using the sensitive EDS detector, there appeared to be some variations in elemental components in those particles (Fig. 4a–d). For example, the magnesium signal in the tiny particles appears to be high (Fig. 4a1–a3,b1–b3). The magnesium peak was evident in the spectral map of the tiny particle although not in that of the large particle (Fig. 4c,d). In the merged intensity maps of calcium and magnesium, which were colored in green and red, respectively, the tiny CaPs had a high magnesium signal overlapping the particle with the calcium signal, whereas calcium but not magnesium was clearly detected in the large CaPs (Fig. 4e–g). These results show that the combination of light and electron microscopic observation as well as HPF-FS and resin embedding is useful for detecting small CaPs and histological ultrastructure in large kidney tissues and suggest the elemental variance of in situ CaPs in kidney tissues produced under HPD.

## Discussion

This study introduced an approach for correlative or sequential light and electron microscopic observation of CaPs, which is also called CaP-CLEM, in kidney tissues. This approach combines fluorescent staining for CaPs and EDS in SEM on resin sections prepared using HPF followed by FS. Light microscopic observation of alendronate conjugated with fluorescent dye and metachromasia in toluidine blue staining indicated the distribution of large CaPs formed under a long-term HPD in the corticomedullary border of the mouse kidney. The presence of CaPs was verified using the correlative or sequential analyses of calcium, phosphorus, and oxygen with EDS on the same or serial sections. Using fluorescent alendronate, small CaPs formed under short-term feeding were also detected, and EDS on serial sections showed that the content of elements, including magnesium, was topographically variable among various particles. These results suggest that CaP-CLEM helps clarify the distribution and elemental CaP composition in kidney tissues and would contribute to understanding the pathophysiology associated with the formation and development of CaPs in the kidney.

This study performed CaP-CLEM on epoxy resin-embedded sections following HPF and FS. Methods of sample preparation affect CaP detection. HPF and FS were selected since they are useful for maintaining morphology and in situ distribution and states of CaPs in the tissues^17,21^. While freeze-thawing promoted the phase transition of amorphous calcium phosphate into crystalline calcium phosphate and increased binding^15^, decreasing the number of freezing and rapid freezing of the tissues, which was followed by treatments in organic solvents, could be better than chemical fixation; this could be the best possible approach. While heavy metal staining typically enhances the visualization of surrounding structures in EM observation, it was observed that the use of common heavy metals for electron microscopic staining, including lead, impaired CaP detection, probably because of the ion exchange mechanism of calcium and lead ions^19^. Therefore, visualizing tissue structures without additional metal staining after FS with organic solvents containing only osmium tetroxide was necessary. In this study, highly sensitive BSE detectors were used to overcome this problem. Additionally, the use of carbon nanotube tape was beneficial for efficient observation of ultrastructure, fluorescence imaging, and subsequent EDS detection without noticeable charging in single sections since the tape is fluorescently transparent, highly conductive to prevent charging, and useful for hydrophilic mounting of the sections^23^. Conversely, one limitation of the approach used in this study was the difficulty of detecting small amounts of carbon in CaPs, since the background signals of carbon, which was considered to be derived from the resins and the carbon nanotube tape, were high. However, combining these technologies was crucial for efficient CaP detection with tissue structures and fluorescence at the electron and light microscopic levels, respectively.

The application of alendronate derivatives conjugated using fluorescent dye successfully detected CaPs on sections of kidney tissues prepared with HPF-FS and embedded in epoxy resin. Moreover, the metachromasia in toluidine blue staining was a prominent phenomenon and may be useful for detecting large CaPs. However, in our experience, metachromasia was unusable for small CaPs, suggesting that metachromasia is a chemical feature of large CaPs. Alternatively, the detailed evaluation of staining conditions in this study suggested that the background fluorescence of CaPs was negligible in the resin sections. Such physical CaP properties facilitated small CaP detection using the fluorescent alendronate derivative. Given that the epoxy resin used in this study is hydrophobic, although the use of hydrophilic resins can alter labeling efficacy in immunoelectron microscopy^24^, it is possible that the optimization of resin formula may increase the efficacy of fluorescent labeling of CaPs on the resin sections. This approach could be applicable to and useful in different organs other than the kidneys when the investigation of small CaPs is needed since the areas observable in electron microscopy for ultrastructural and elemental analyses are very limited.

The CaP-CLEM in this study suggested the differential elemental composition, including magnesium, in CaPs produced in kidney tissues under HPD. CaP formation could be associated with variable phases of calcium phosphate, which has different calcium-to-phosphate molar ratios and water solubility and may involve the incorporation of other ions^25,26^. Sufficient evidence suggests that magnesium inhibits the formation and growth of hydroxyapatite^6,27,28^. Furthermore, magnesium has been suggested to have a direct effect on cellular signaling to prevent CaP development in the in vitro model of vascular calcification^10^. Our results suggest that magnesium is also incorporated in the small CaPs in the kidney but somehow decreased in larger CaPs. It supports the hypothesis that substantial incorporation of magnesium in CaPs can facilitate the maintenance of the small CaP size. The small CaPs with magnesium signals may also be associated with the distribution of amorphous CaPs, which could be stabilized by magnesium^6,10^. CaP-CLEM could be useful for examining potential mechanisms and roles of magnesium involvement in CaP formation and development under increased phosphate excretion in renal tubules in vivo.

## Materials and methods

### Animal experiments

Animal housing and HPD feeding were conducted as previously described^4^. Briefly, four wild-type C57BL/6 mice were placed on a diet containing 2.0% inorganic phosphate for 10 days or 4 weeks. The other four mice were placed on a normal diet for the same periods for control. Experiments were approved by the Institutional Animal Care and Use Committee and performed under the guidelines on the care and use of animals at Jichi Medical University. The animal experiments were performed in accordance with the Animal Research: Reporting In Vivo Experiments (ARRIVE) guidelines. All efforts were made to use only the number of animals necessary.

### Tissue collection, fixation, and embedding

At the end of the feeding, kidney tissues of mice fed with an HPD or normal diet were obtained following euthanasia, cut into small pieces in Krebs–Ringer HEPES buffer, and fixed with either immersion fixation or HPF using EM ICE (Leica). In the immersion fixation, some pieces of kidney tissues were immersed in buffered formalin, embedded in paraffin wax, and observed using von Kossa staining after sectioning, as previously described^20^. The frozen tissues were embedded in epoxy resin as previously described^4^. Briefly, the tissues were freeze-substituted in acetone containing 2% osmium tetroxide (Nissin EM) using EM AFM2 (Leica). The substitution medium was replaced with pure acetone after increasing the temperature and incubating at room temperature, and the samples were embedded in Quetol 812 epoxy resin (Nisshin EM Co.).

### Light and electron microscopy

The samples in the cured resin were sectioned at 500-nm thickness. Some of the serial sections were mounted on glass slides, stained with toluidine blue or different concentrations of the alendronate conjugated with FITC or Cy3^15^, and imaged using a light microscope (AX80, Olympus). To detect small and weak signals, some of the fluorescence images were handled and processed using Fiji (https://imagej.net/software/fiji/) for background subtraction and linear contrast stretching. The other serial resin sections were mounted on pieces of conductive carbon nanotube tape^23^, which has several advantages in this study, including little fluorescence background, well-preserved hydrophilicity, and high electron conductivity. The sections on the tape were imaged in SEM with or without prior staining of alendronate conjugated with Cy3. The SEM imaging was performed using S-4300 (Hitachi) equipped with an EDS detector, EMAX-MODEL6853-H (Horiba), or Scios2 (Thermo Fisher Scientific) equipped with retractable annular BSE detector and an EDS detector (Ultim Max 170, Oxford Instruments). In Scios2, the imaging was performed in similar conditions previously reported^4^. The secondary electron and backscatter electron images were obtained at an accelerating voltage of 5 kV and 0.4-nA probe current, and EDS images were acquired at an accelerating voltage of 10 kV and 0.8-nA probe current.

## Data Availability

The datasets used and/or analysed during the current study are available from the corresponding author on reasonable request.
